# Psychological Resilience Mediates the Impact of Childhood Trauma on Depressive Symptoms in Major Depressive Disorder

**DOI:** 10.3390/jcm14051516

**Published:** 2025-02-24

**Authors:** Mehmet Baltacioğlu, Meltem Puşuroğlu, Bülent Bahçeci, Begüm Aydın Taslı, Burak Okumus

**Affiliations:** 1 Department of Psychiatry, Faculty of Medicine, Recep Tayyip Erdoğan University, Rize 53100, Turkey; meltempusuroglu@gmail.com (M.P.); bulentbahceci@hotmail.com (B.B.); baydin114@gmail.com (B.A.T.); 2 Department of Psychiatry, Faculty of Medicine, Usak University, Usak 64200, Turkey; okumusband@gmail.com

**Keywords:** major depressive disorder, depressive symptoms, childhood trauma, mediating effects, psychological resilience

## Abstract

**Background:** This research aimed to investigate the mediating and moderating effects of psychological resilience (PR) on the association between childhood trauma (CT) and the development of depression. **Methods:** This study included 94 cases who consecutively applied to the outpatient psychiatry clinic of Rize Recep Tayyip Erdoğan University Training and Research Hospital in Turkey between 1 June 2023 and 1 December 2023 and were diagnosed with Major Depressive Disorder (MDD). In addition, 83 healthy individuals (control group) were also included. Participants administered the Hamilton Depression Rating Scale (HDRS), the Resilience Scale for Adults (RSA), and the Childhood Trauma Questionnaire (CTQ). **Results:** In the context of this research, the mediating effect of PR between CT and depression development was investigated. When examining the mediating role of PR on the association between CT and depression, it was found that CT statistically significantly influenced disease severity directly (B = 0.158, SE = 0.0398, z = 3.98, *p* < 0.001, 95% CI [0.0759, 0.232]), while it also influenced disease severity indirectly through PR (B = 0.193, SE = 0.028, z = 6.88, *p* < 0.001, 95% CI [0.1343, 0.244]). It was revealed that PR mediated the association between CT and depressive symptoms partially. **Conclusions:** This study demonstrates that PR has a mediating effect on the relationship between CT and MDD. These results provide new contributions to the existing literature. Interventions that increase psychological resilience may positively contribute to the treatment of patients with MDD who have suffered from CT.

## 1. Introduction

Major Depressive Disorder (MDD) is a recurrent mood disorder defined by persistent sadness, loss of interest, and a lack of energy. Its lifetime prevalence ranges from 2.2% to 26.8%. It is related to high mortality, morbidity, and disability [1]. According to the World Health Organization (WHO) 2008 data, MDD ranks third among all medical diseases that cause disability. This ranking is expected to rise to first place by 2030 [2]. The etiology and pathogenesis of MDD have not yet been fully elucidated. Related studies have focused on the gene–environment interaction. It is believed that childhood trauma (CT) has a significant place among environmental factors [3].

CT profoundly affects both physical and mental health, leaving lasting and indelible marks on individuals’ lives [4]. It can manifest as physical, emotional, and sexual neglect and abuse [5]. WHO (2019) reports that 75% of children are exposed to trauma by their parents or caregivers. The WHO (2019) also reports that 23% of these children experience physical abuse, 36% experience emotional abuse, 18% sexual abuse, and 16% physical neglect [6,7]. A study found that approximately 45% of children in the U.S. had at least one traumatic experience in their lifetime, and some experienced multiple traumatic events [8]. It is claimed that exposure to traumatic experiences can elevate the risk of mental disorder development in adulthood [9]. It is stated that childhood trauma, especially when experienced in the early years, is linked to an increased prevalence of various mental disorders, including MDD, post-traumatic stress disorder, dissociative disorders, sexual dysfunction, personality disorders, and substance use disorders [10,11,12]. One study found that CT increased the risk of developing MDD in adulthood 2.6 times [13]. Another study reported that about 50% of individuals with a CT history were diagnosed with MDD in adulthood [14]. Studies investigating CT in patients with MDD have encountered similar results [7,15,16]. In a meta-analysis of 6830 MDD patients, Kuzminskaite et al. found that approximately 62% of these patients had a history of CT [9]. In terms of the subtypes, the most common were emotional neglect (38.9%) and abuse (24.8%). It was shown that frequent exposure to trauma was related to negative life events such as cognitive impairment, suicidal tendencies, and sleep problems [17].

However, it has been noted that not all traumatic experiences result in mental disorders. This situation is associated with psychological resilience (PR) [18]. PR is the capacity to adapt to traumatic experiences. Additionally, as a personality trait, it is used in the sense of overcoming difficulties, resilience, flexibility, and robustness [19]. Another point here is the affective temperaments that have an effect on affective regulation. They can also increase the resilience of individuals against trauma, just like PR. For example, Favaretto and colleagues emphasize that affective temperaments play an important role in psychiatric disorders. They suggest that hyperthymic temperament, in particular, can interact with resilience mechanisms and prevent the development of depression after CT [20]. In addition, PR may reduce the effects of trauma by helping individuals regulate their emotional responses in stressful situations, develop cognitive flexibility, and strengthen their social support networks [20,21]. From a neurobiological perspective, PR may prevent excessive stress responses after trauma by contributing to the regulation of the hypothalamic–pituitary–adrenal (HPA) axis and increasing the control of the prefrontal cortex over the amygdala [22]. In addition, it is known that PR supports neuroplasticity and helps repair neural damage caused by trauma [23]. In therapeutic processes, interventions that strengthen PR (e.g., cognitive–behavioral therapy, psychoeducation, social support, exercise) support individuals’ post-traumatic recovery processes and can improve emotion processing skills [24,25]. Considering all these, the effect of PR on the relationship between CT and the development of depression has become a matter of curiosity. Research in the literature has revealed that PR is associated with the onset of mental disorders, the clinical course of it, and the quality of life [26]. Moreover, it is stated to have a protective role against mental disorders [27]. Indeed, in a study focusing on the association between mental health and resilience, Hu et al. found that PR plays a protective role against mental illness [15]. In a study comparing individuals who experienced emotional neglect in childhood in terms of resilience, Campbell-Sills et al. stated that individuals whose resilience levels were high reported fewer mental symptoms than individuals who had low resilience levels [28]. In another study, Schulz et al. linked both psychological resilience and CT to adult depression. They also paired low resilience with the devastating effects of depression [18].

Although many studies have extensively examined the association between CT and depression, other factors involved in this relationship have not been sufficiently explored. In particular, the impact of protective mechanisms such as PR on this process have not been understood fully. Though recent studies have turned their attention to this area, there is still a lack of sufficient research. The purpose of this study was to investigate the relationship between CT and the development of depression and to examine the mediating and moderating roles of PR in this relationship. It was hypothesized that CT would have negative impacts on the development of depression and that PR would have a mediating and moderating role in this relationship. In this study, PR was chosen as the focus, and it was planned to better understand the effectiveness of protective mechanisms in the relationship between CT and the development of depression and to include interventions aimed at psychological resilience in the treatment protocols.

## 2. Materials and Methods

### 2.1. Participants

This study included 94 patients who consecutively applied to the psychiatry outpatient clinic of Rize Recep Tayyip Erdoğan University Training and Research Hospital in Turkey between 1 June 2023 and 1 December 2023 and were diagnosed with MDD according to the DSM-5 criteria. In addition, a control group of 83 healthy individuals who had comparable sociodemographic characteristics was included in this study. Power analysis was performed using G*Power software (latest Ver. 3.1.9.7; Heinrich-Heine-Universität Düsseldorf, Düsseldorf, Germany) with 5 statistical examples, and at a 60% effect size, 0.05 type 1 error (α) and 95% power level, the case and control groups were evaluated at a distribution ratio of 1.5, and the minimum number of people to participate in this study was calculated as 148. This study was completed with a total of 177 people. This study included patients diagnosed with MDD who were between the ages of 18–65, literate, able to understand what they read, and volunteered to participate in this study. Individuals who were illiterate, under the age of 18, over the age of 65, pregnant or breastfeeding, had significant psychotic symptoms, comorbid neurological diseases, psychiatric disorders, alcohol and substance addiction, intellectual disability, conditions that prevent communication, and did not volunteer to participate in this study were excluded from this study. The control group, which consisted of patient relatives and healthcare professionals with sociodemographic characteristics matched with the patient group, included individuals who did not have any mental illness, intellectual disability, or alcohol or substance addiction.

### 2.2. Ethics Approval

All participants received comprehensive information regarding this study’s objectives and procedures; subsequently, written informed consent was obtained from each of them. Prior to this study, the Faculty of Medicine’s Non-Interventional Clinical Research Ethics Committee at Recep Tayyip Erdoğan University provided ethical approval (Ethics Approval No: E-40465587-050.01.04-707; date: 26 May 2023; Decision No: 2023/127). This research study was carried out in accordance with the ethical guidelines outlined in the 1964 Declaration of Helsinki. Throughout this research study, the ethical principles of corporate and/or national research committees were followed.

## 3. Data Collection Instruments

### 3.1. Sociodemographic Data Form

This instrument was developed by the researcher regarding the specific characteristics of this study and was used to reveal the sociodemographic and clinical characteristics of individuals. In short, this form was utilized to investigate the gender, age, occupation, education level, socioeconomic status, and clinical course of the disease of the individuals.

### 3.2. Hamilton Depression Rating Scale (HDRS)

The HDRS was created by Max Hamilton in 1960 to measure the severity of depression [29]. The scale, initially developed by Hamilton as a 17-item scale, has been expanded to include 21 and 24 items in subsequent years. In this study, the 17-item form was used. The maximum possible score that can be reached on the scale is 53 [30]. Akdemir and colleagues carried out a study to assess the reliability and validity of the scale in the Turkish context. Akdemir et al. calculated the scale’s Cronbach’s alpha value as 0.75 [31]. In this study, on the other hand, we calculated this value as 0.77.

### 3.3. Psychological Resilience Scale for Adults (RSA)

The RSA was created by Friborg et al. in 2003 to measure psychological resilience (PR) [32]. The scale, developed as a five-factor model in 2003, was revised in 2005 to include six factors: “*perception of self*”, “*planned future*”, “*social competence*”, “*structured style*”, “*family cohesion*”, and “*social resources*”. The instrument comprises 33 items, each of which is evaluated on a 5-point Likert scale. Scores on this instrument are continuous, with no predetermined point defining a specific level of resilience. Higher scores generally reflect a stronger capacity for psychological resilience [33]. Basım and Çetin [34] adapted the scale for use in the Turkish population. While the instrument’s Cronbach’s alpha was calculated as 0.86 in the study of Basım and Çetin, we determined this statistic as 0.82 in our study.

### 3.4. Childhood Trauma Questionnaire (CTQ)

The CTQ is a 28-item self-report scale created to retrospectively evaluate quantitative abuse and neglect issues experienced prior to the age of 20. It comprises 5 factors: “*emotional abuse*”, “*physical abuse*”, “*sexual abuse*”, “*emotional neglect*”, and “*physical neglect*”. The instrument was originally created by Bernstein et al. in 1995 [35]. It was adapted for the Turkish population and validated by Şar et al. The scale’s Cronbach’s alpha value was reported as 0.93 in that research [36]. In our study, on the other hand, this value was found to be 0.89.

## 4. Statistical Analysis

The normality of the distribution of HDRS score, CTQ and its subscale scores, and RSA and its subscale scores across groups was evaluated by utilizing the Kolmogorov–Smirnov test. To compare age, number of children, years of education, HDRS score, and RSA subscales’ scores that did not distribute normally across groups, the Mann–Whitney U test was applied. An independent-samples *t*-test was used to compare the total RSA score that had a normal distribution across groups. In addition, mediation analysis was conducted using the Bootstrap technique to examine the effect of one measurement tool on another through a moderator. The analysis results were shown as mean, standard deviation (Mean ± SD), minimum (min), maximum (max), and median for quantitative data and as frequency (n) and percentage for categorical data. In all calculations and interpretations, results were considered statistically significant when the *p*-value was less than 0.05. Data were analyzed utilizing SPSS (IBM Corp., Armonk, NY, USA, Version 27) and Jamovi (The jamovi project, 2023) programs.

## 5. Results

**Figure 1:**
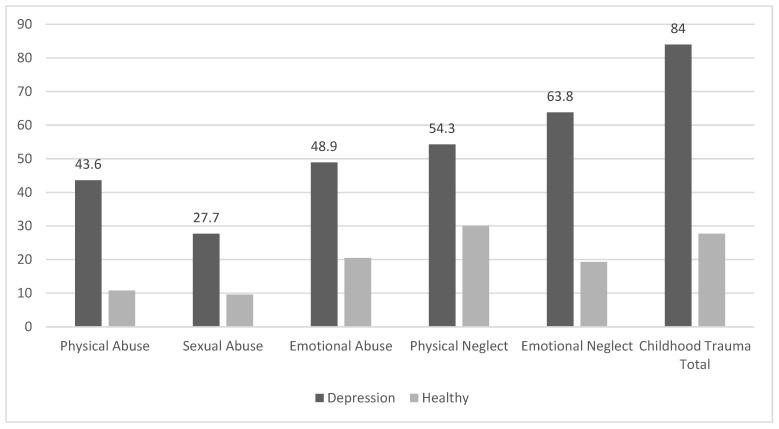
CT rates based on cutoff scores of the scale scores in the experiment and control groups.

A total of 177 individuals, 94 of which were in the patient group and 83 in the control group, were included in this study. A total of 68.1% of the patient group (n = 64) were female and 31.9% (n = 30) were male; 33.7% of the control group (n = 28) were male, while 66.3% were female (n = 55). On average, participants in the patient group were 32.85 years old with a standard deviation of 10.59 years. The control group had a mean age of 33.06 years with a standard deviation of 10.56 years. In the patient group, 47.9% (n = 45) were single and 42.6% were married (n = 40); 27.7% of the patient group (n = 26) were employed, and 61.4% of the control group (n = 51) were employed. No statistically significant disparities were determined between the patient and healthy (control) groups for age, number of children, educational level, gender, and marital status (*p* > 0.05). On the other hand, a statistically significant divergence was observed in the occupational profiles of the patients across the groups (*p* < 0.001), and this difference was determined in patients who were currently employed, unemployed, and students. The proportion of healthy individuals who were currently employed was higher compared to those with depression. The distribution of sociodemographic and clinical characteristics of patients (experiment) and the control group is presented in Table 1 and Figure 1.

Comparing the scores obtained from the instrument for the experiment and control groups, it was determined that the total scale and subscale scores (*physical abuse*, *sexual abuse*, *emotional abuse*, *physical neglect*, and *emotional neglect*) of the CTQ were statistically significantly higher in the experiment group compared to the control group (*p* values were *p* < 0.001, *p* < 0.001, *p* < 0.001, *p* < 0.001, *p* < 0.001, and *p* < 0.001, respectively). Conversely, it was observed that the total scale score and scores of all subscales (*structural style*, *planned future*, *family cohesion*, *perception of self*, *social competence*, *and social resources*) of the RSA were statistically significantly higher in the control group compared to the patient group (*p* < 0.001). The comparison of the scale scores of the experiment and control groups is presented in Table 2.

**Figure 2:**
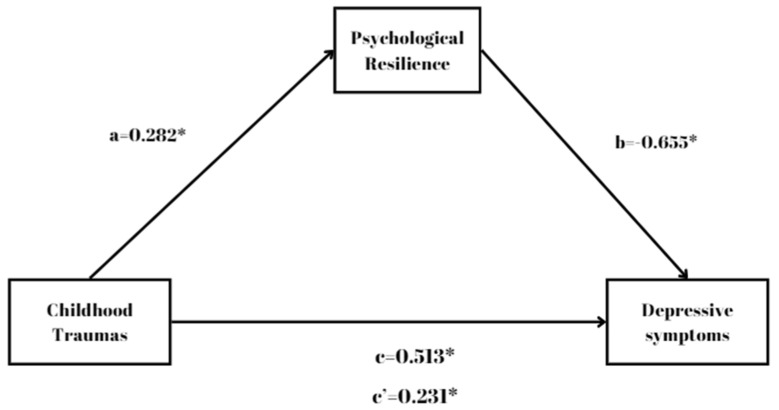
Findings regarding the mediating role of PR in the association between CT and disease severity. * *p* < 0.001, a: Effect of childhood traumas (CTQ) on psychological resilience (RSA). b: Effect of psychological resilience (RSA) on disease severity (HDRS). c’: Effect of childhood traumas (CTQ) on direct disease severity (HDRS). c: Total effect of childhood traumas (CTQ) on disease severity (HDRS). Childhood trauma (CT): independent variable. Depressive symptoms (DSs): dependent variable. Psychological resilience (PR): mediator variable. In this way, the direct effect of CT on DS and the mediator role of PR in this effect were evaluated.

When the mediating role of PR in the association between CT and depressive symptoms was examined, it was found that the indirect effect of CT on disease severity through PR was significant (B = 0.193, SE = 0.028, z = 6.88, *p* < 0.001, 95% CI [0.1343, 0.244]). This means that the confidence interval for the regression coefficient does not include 0. The effect of CT on PR (B = −0.784, SE = 0.1126, z = −6.97, *p* < 0.001, 95% CI [−0.9898, −0.549]) and the impact of PR on disease severity (B = −0.246, SE = 0.016, z = −15.4, *p* < 0.001, 95% CI [−0.2772, −0.215]) were also found to be statistically significant. The direct impact of CT on disease severity was also significant (B = 0.158, SE = 0.0398, z = 3.98, *p* < 0.001, 95% CI [0.0759, 0.232]). In conclusion, the total impact of CT on disease severity (B = 0.351, SE = 0.0535, z = 6.56, *p* < 0.001, 95% CI [0.2381, 0.448]) was determined as statistically significant. In addition, it was observed that the impact of CT on disease severity was statistically significant, both directly and indirectly through PR. The results reveal that the relationship between CT and disease severity appears to be partially mediated by PR, as shown in Figure 2 and Table 3.

## 6. Discussion

This study aimed to investigate the mediating and moderating impacts of PR on the relationship between CT and the development of depression. Our study revealed that the total and subscale scores of the CT scale and the RSA were statistically significantly higher in patients with MDD compared to the control group. Additionally, it was seen that CT was associated with the development of depression both directly and through PR. It was determined that PR mediated this relationship both directly and partially. It was found that traumatic experiences such as neglect and abuse experienced in childhood can heighten the risk of depression in adulthood, while high PR can decrease this risk.

CT is frequently observed in MDD [37,38]. The literature suggests that traumas experienced early in life enhance the risk of developing MDD in adulthood, impact the course of the disorder negatively, and delay recovery [9]. It has been observed that in particular, early emotional neglect and abuse are related to an increased risk of depression [5]. In a study examining the prevalence of CT in MDD, Goldberg et al. revealed that approximately 57% of patients diagnosed with MDD in adulthood had experienced trauma in childhood [39]. In another study, this rate was found to be 46% [40]. A larger-scale meta-analysis study reported that approximately 45.59% of patients diagnosed with MDD had experienced CT at some point in their lifetime, and 19.13% had experienced more than one CT. It was determined that 25.27% of the experienced traumas were sexual abuse, 27.59% were physical abuse, 36.72% were emotional abuse, 43.2% were emotional neglect, and 36.18% were physical neglect. Moreover, this study indicated that there was a significant association between experienced emotional abuse and neglect and both an earlier age of disorder onset and treatment resistance [40]. Similar results were also obtained in the comparisons performed with the control group by using the scale scores. It was reported that MDD patients had higher scores on CT scales compared to the control group [16,41]. In a research study where Kaczmarczyk et al. compared 68 MDD patients with 75 healthy controls in terms of CT, it was found that compared to healthy individuals, individuals with MDD exhibited significantly higher total CT scale scores [41]. Consistent with the literature, we also found that in patients with MDD, both the prevalence of CT and the total CT scale scores were significantly higher than the individuals in the control group. In addition, this result is consistent with the results of two other studies conducted in our country. Similarly to our study, these studies also found that CT rates and scale scores were higher in patients with MDD than in healthy controls [10,16]. PR, defined as the ability to adapt to threatening and stressful situations, is a protective mechanism for the prevention of the development of many mental disorders. It is also crucial for psychosocial functioning [4]. The association between resilience and depressive symptoms has been shown in numerous studies [15,42,43]. Studies on this topic have revealed a negative association between resilience and depressive symptoms. They have reported a link between low resilience and heightened levels of both depression and anxiety [15]. Similarly, research has demonstrated that individuals with high resilience levels tend to report fewer anxiety and depressive symptoms after stressful life events or negative life experiences, such as childhood trauma, compared to individuals with low resilience [44]. This result was also supported by a study conducted in our country. In a study conducted by Bilican et al., high psychological resilience was similarly associated with psychological well-being and low depression frequency [45]. In a study that assessed resilience in patients with MDD, Maekawa et al. determined that resilience scores were higher in the healthy group than in MDD patients, similar to previous studies [46]. In the current research, similar to the study of Maekawa et al., it was observed that RSA scores were higher in the control group than in MDD patients. Furthermore, this result was consistent with the study of Seok et al. [47].

Depression is a complex condition arising from the interaction of numerous factors, including environmental and genetic factors [48]. Among these factors, the impact of CT is particularly significant. Research has revealed that individuals with past CT experiences have a higher risk of developing depression than those who have not [40,49]. Moreover, it has been shown that CT is a significant predictor of the onset of long-term depression [50]. However, it has been reported that not every traumatic experience results in depression [18,51]. This has led researchers to focus on mediating and protective mechanisms such as family support, social support, and psychological resilience. Among these mechanisms, psychological resilience has received the most attention [52]. Especially in studies conducted with children and adolescents, it has been revealed that psychological resilience is a mediating and protective factor in the association between adult depression and CT [53,54]. Similar findings have been observed in studies conducted with adults [18,50,55]. In a study evaluating 792 adults who had a trauma history, Wingo et al. determined that psychological resilience played a mediating and moderating role in the development of depression among individuals with a CT history. Furthermore, they reported that exposure to CT increased depressive symptoms, while resilience decreased these symptoms [55]. Similarly, Schulz et al. reported that traumatic experiences such as neglect and abuse experienced in childhood increased the risk of developing adult depression, while resilience reduced the harmful effects of these traumatic experiences in childhood [18]. In a different study, Zheng et al. investigated the mediating effects of both daily stressors and psychological resilience. They found that CT directly and indirectly mediated the onset of depression through daily stressors and psychological resilience [50]. A meta-analysis study carried out recently by Zhao et al. also yielded similar results. In that study focusing on 33 related research, the authors stated that psychological resilience acts as a mediating and moderating factor in the association between CT and depression, both directly and by mitigating the effects of CT [56]. Consistent with previous studies, in the current study, we determined that CT had an impact on the development of depression and that psychological resilience had a partial and mediating role in this effect.

Psychological resilience has significant effects on the course of the disease, symptom severity, level of functionality, response to treatment, and the person’s quality of life. It has been shown that adverse childhood experiences and coping methods used are important in determining the level of psychological resilience [18]. Although there are few studies examining the relationship between the level of psychological resilience and the decrease or improvement in symptom severity in mental illnesses, the current findings show that the level of psychological resilience is associated with recovery, regardless of the severity of the illness [57].

We can also talk about the limitations of this study. This study’s limitations include its single-center setting, small sample size, and cross-sectional design. Additionally, the exclusion of confounding factors such as medication use, age, and duration of disease can also be considered a limitation. Another limitation is that CT, which is among past experiences, was assessed using self-report scales, and the mediation model was based on total scores. The use of self-reported assessment tools in the assessment of psychological resilience can also be considered as a limitation of our study. Because individuals may have interpreted some scale items differently. However, there is no tool that can be used to objectively assess the psychosocial determinants of the level of psychological resilience. PR is a dynamic process that can change throughout life. The fact that the difficulties or stressful events experienced by individuals in daily life were not taken into account in determining the levels of psychological resilience in the post-treatment follow-up was considered a confounding factor in the evaluation of post-treatment psychological resilience levels and this was interpreted as a limitation of our study.

While this study has some limitations, it also has several strengths. Although the association between CT and mental disorders has been examined in numerous studies, the impact of PR on this relationship has been investigated in a limited body of research. The fact that this is one of the few studies conducted in this area can be considered a strength of this study. Additionally, the fact that this study was carried out with a control group and that the data used in this study were obtained through standardized scales can be shown as the other strengths of this study.

## 7. Conclusions

This study revealed a high prevalence of CT in patients diagnosed with MDD. It was observed that increasing CT was related to the development of depression both directly and through protective mechanisms such as PR. PR was found to have a significant mediating and moderating effect on this relationship. It was determined that increased traumatic experiences enhanced the risk of depression, while protective factors such as PR decreased this risk. Enhancing the effectiveness of protective mechanisms like PR, which is a significant factor in the development of depression with CT, is crucial for the prevention of future mental pathologies. In the future, there will be a need for training and support programs that will increase the effectiveness of such protective mechanisms. Especially in children growing up in high-risk families, identifying risk situations and activating protective mechanisms early will be crucial for preventing negative mental pathologies in the future.

In conclusion, integrating techniques that increase PR, such as cognitive–behavioral therapy, mindfulness-based stress reduction practices, regular exercise, social support, and psychoeducation, into treatment processes may be effective in preventing the development of mental pathologies in individuals exposed to trauma. These approaches may help individuals preserve their mental health in the long term by strengthening their coping skills with traumatic experiences. However, in order to better understand these effects, future studies with larger samples and longitudinal studies that cover different cultural and socioeconomic groups and examine the effects of different subtypes of CT (such as emotional, physical, sexual abuse, and neglect) are needed.

## Figures and Tables

**Table 1:** Distribution of sociodemographic and clinical characteristics of the experiment and control groups.

Variable	Depression		Healthy		Test Stat.	*p*
Age	32.85 ± 10.59	32 (18–60)	33.06 ± 10.56	32 (18–58)	−0.156	0.876 ^m^
Number of children	1.14 ± 1.41	0 (0–4)	1.05 ± 1.3	0 (0–5)	−0.342	0.732 ^m^
Education (Year)	12.68 ± 4.31	14 (5–18)	13.3 ± 3.96	14 (5–20)	−0.730	0.466 ^m^
Gender						
Female	64 (68.1)		55 (66.3)		0.066	0.797 ^pc^
Male	30 (31.9)		28 (33.7)			
Marital status						
Single	45 (47.9)		36 (43.4)		2295	0.328 ^f^
Married	40 (42.6)		43 (51.8)			
Divorced	9 (9.6)		4 (4.8)			
Occupation						
Employed	26 (27.7) ^a^		51 (61.4) ^b^		21,186	<0.001 ^f^
Unemployed	36 (38.3) ^a^		16 (19.3) ^b^			
Retired	2 (2.1) ^a^		2 (2.4) ^a^			
Student	30 (31.9) ^a^		14 (16.9) ^b^			
Hospitalization in Psychiatric Clinic						
Yes	7 (7.4)		---		---	
No	87 (92.6)		---			
Number of Hospitalizations in Psychiatric Clinics	8.98 ± 4.18	7 (5–23)	---			
Age of First Disease	26.03 ± 7.3	24 (16–57)	---			
Number of Depressive Episodes	1.91 ± 1.08	2 (1–6)	---			

^m^: Mann–Whitney U test, ^pc^: Pearson chi-square test, ^f^: Fisher’s exact test, ^a,b^: Groups designated by the same letter exhibit no significant variations (Z test with Bonferroni correction), mean ± SD, median (min.–max.), n (%).

**Table 2:** Comparison of scale scores between the experiment and control groups.

Variable	Depression		Healthy		Test Stat.	*p*
Physical Abuse	6.99 ± 3.71	5 (5–25)	5.29 ± 1.02	5 (5–12)	−4.880	<0.001 ^m^
Sexual Abuse	5.86 ± 1.96	5 (5–17)	5.18 ± 0.75	5 (5–11)	−3.213	<0.001 ^m^
Emotional Abuse	8.98 ± 4.18	7 (5–23)	5.92 ± 1.77	5 (4–15)	−6.357	<0.001 ^m^
Physical Neglect	8.74 ± 2.94	8 (5–18)	6.78 ± 2.39	6 (5–15)	−5.214	<0.001 ^m^
Emotional Neglect	13.83 ± 4.89	14 (5–25)	9.22 ± 3.68	9 (5–18)	−6.214	<0.001 ^m^
Childhood Trauma Total Score	44.94 ± 12.15	43 (26–88)	32.61 ± 6.72	31 (25–54)	−7.745	<0.001 ^m^
Structural Style	11.04 ± 3.24	11.5 (4–19)	13.63 ± 3.61	14 (8–20)	−4.429	<0.001 ^m^
Planned future	10.53 ± 3.64	11 (4–19)	13.69 ± 3.88	13 (8–20)	−4.988	<0.001 ^m^
Family Cohesion	12.93 ± 5.49	11 (5–27)	20.98 ± 5.38	21 (12–30)	−7.995	<0.001 ^m^
Perception of Self	14.3 ± 4.84	14 (7–28)	21.05 ± 5.72	21 (12–30)	−7.096	<0.001 ^m^
Social Competence	14.96 ± 4.4	14 (7–25)	20.59 ± 5.51	20 (12–30)	−6.428	<0.001 ^m^
Social Resources	16.05 ± 3.97	16 (9–25)	25.3 ± 6.07	26 (13–35)	−8.860	<0.001 ^m^
Psychological Resilience Total	79.81 ± 11.43	79 (57–110)	115.23 ± 12.55	114 (84–140)	−19.651	<0.001 ^t^

^m^: Mann–Whitney U test, ^t^: independent two-sample *t* test, mean ± SD, median (min.–max.).

**Table 3:** Examining the mediating impact of PR on the association between CT and disease severity (Hamilton Depression Rating Scale).

Type	Effect	B	SE	β	z	*p*	95% Confidence Interval	
							Lower Limit	Upper Limit
Indirect effect	CTQ -> RSA -> HDRS	0.193	0.028	0.282	6.88	<0.001	0.134	0.244
Components	CTQ -> RSA	−0.784	0.1126	−0.43	−6.97	<0.001	−0.989	−0.549
	CTQ -> HDRS	−0.246	0.016	−0.655	−15.4	<0.001	−0.277	−0.215
Direct effect	CTQ -> HDRS	0.158	0.0398	0.231	3.98	<0.001	0.075	0.232
Total	CTQ -> HDRS	0.351	0.0535	0.513	6.56	<0.001	0.238	0.448

B: coefficient; β: standardized coefficient; SE: standard error; CI: confidence interval; CTQ: Childhood Trauma Questionnaire; RSA: Psychological Resilience Scale.

## Data Availability

Data associated with this research are available upon request from the corresponding authors.

## Abbreviations

PR	Psychological resilience
CT	Childhood trauma
MDD	Major Depressive Disorder
HDRS	Hamilton Depression Rating Scale
RSA	Resilience Scale for Adults
CTQ	Childhood Trauma Questionnaire
WHO	World Health Organization
DSM-5	Diagnostic and Statistical Manual of Mental Disorders
